# Pregnant Teenager Involvement in Sexual Activity and the Social Context

**DOI:** 10.1100/tsw.2006.188

**Published:** 2006-08-25

**Authors:** Maria José Carvalho Sant'Anna, Júlia Kerr Catunda, Kepler Alencar Mendes Carvalho, Veronica Coates, Hatim A. Omar

**Affiliations:** ^1^ Adolescent Clinical Unit, Department of Pediatrics, Faculty of Medical Sciences, Santa Casa, São Paulo, Brazil; ^2^ Department of Pediatrics, University of Kentucky, Lexington, KY, USA

**Keywords:** adolescent sexual behavior, predicting sexual initiation, risk factors, Brazil

## Abstract

Pregnancy during adolescence represents a challenge to society as a whole. Its incidence is increasing and brings about social and medical consequences to both the teen mothers and their children. The purpose of this study was to evaluate pregnant teenager involvement in sexual activity and the social context. The group studied comprised 152 pregnant teenagers attending the Department of Pediatrics, Santa Casa de Sao Paulo (SCSP) General Hospital. All information was analyzed. The age at first intercourse was 14.2 years and the average period between first intercourse and pregnancy was 1.4 years. Most pregnancies (75%) were neither planned nor wanted, however, most teen mothers (64.3%) did not use any contraceptive method. Of the pregnant teenagers, 68.1% came from unstructured families where in 71% of the teen pregnancy cases, there was a role model (mother, sister, or cousin who already experienced teen pregnancy). The average number of school years attended by the analyzed pregnant teenagers was 8.1 years, however, there was a high dropout rate of 40.1%. The age at first intercourse was low and concurs with the high incidence of unstructured families. The average number of school years attended was high, which would theoretically reflect a greater knowledge with regard to human reproduction, pointing to the multicausality of teen pregnancy and the role played by the family. Conclusions: We confirmed that teen pregnancy presents multicausal etiology; sexual initiation of pregnant teenagers was quite early with high dropout rates, which indicated that prevention methodology should be based on early detection of risk factors for elaboration of appropriate prevention proposals.

## INTRODUCTION

Teenage involvement in sexual activity without responsibility can constitute risk in various degrees to the life project of the teenager including unwanted consequences, such as sexually transmitted diseases (STDs) and AIDS in particular, undesired pregnancy, and abortion. For teenagers, the birth of a baby or an abortion occurs in a period of intense transformation and, therefore, it is increasingly clear that teen pregnancy is primarily a social problem that can bring about medical consequences[1]. Additionally, there are severe biological, familial, emotional, and economically related implications that impact the pregnant teen individually and society as a whole, which limit or even adjourn the possibility of development and professional engagement of those pregnant teenagers in society[2]. As a result of the consequences for the mother and the conceptus, teen pregnancy has been considered high-risk pregnancy by the World Health Organization (WHO)[3,4]; however, it is currently claimed that the risks are more social than biological. The number of childbirths in the adolescent population corresponds to the about 10% of the total of world births a year; but in Brazil, the number of adolescent mothers' newborns (NB) corresponds to 26.75% of the births, having regional variations with larger taxes in the North and Northeast. The last Brazilian census (2000) showed that in the last 10 years, the fecundity between 10 and 14 years grew 108%. Approximately 40% of the accomplished abortions happened in 20-year-old or younger minors, and according to the data from the Ministry of Health (Brazil), the mortality coefficient due to abortion was the third cause of maternal death[5]. According to Crespin[6], who followed pregnant teenagers in a private clinic for a period of 15 years, 86.2% of the pregnant teenagers opted for induced abortion. Several factors contributed to the occurrence of pregnancy during teenage years, and the etiology was related to biological, familial, social, psychological, and contraception-related factors[7]. Among the biological factors, the increasingly earlier puberty and age of menarche has contributed to the earlier occurrence of sexual activity and the consequences thereof[8]. Influence of the family in the sexual behavior of teenagers has been analyzed under various viewpoints, as the family context has a direct relationship for the time when sexual activity is initiated[9].

The adolescent's home and family atmosphere is considered a risk factor for precocious pregnancy, concluding that the parents' proximity can be an important factor for transmission of knowledge, faiths, and attitudes[10]. Teenagers whose mothers or sisters became pregnant during their teenage years face a risk that is considerably higher to become pregnant themselves[13]. Social factors and ambiguous attitude of society with regard to sexual activity of the teenager leads to conflicting beliefs in the adolescent life. At the same time that society expresses disapproval of early sexual initiation, there exists constant stimulus to eroticism[11]. Some papers demonstrate that religion plays an important part in the development of sexual attitude since many teenagers who are engaged in religious activities acquire a value system that encourages them to develop responsible sexual behavior[12].

Pregnancy in the teen years has direct consequences on the teenage mother, on the teenage father, and on the conceptus. The consequences for the teenage mother include obstetric complications, nutritional disorders, and possibly also psychological and educational problems[13]. There are reports indicating that obstetric complications occur in a greater proportion to pregnant teenagers in the lower age brackets. There has been confirmation of cases of anemia, insufficient weight gain, hypertension, urinary tract infection, STDs, cephalopelvic disproportion, and puerperal complications[14,15]. However, one should be careful to remember that those findings also relate to prenatal care and when there is appropriate prenatal follow-up, the risk is not greater when the same socioeconomic level adult women and teenagers are compared to each other[16,17]. Nutritional consequences are expected to be greater when the pregnancy is closer to menarche because in this period, the pregnant teenager may still be in her growth process. The mother's growth can be impacted as there is extra demand for fetal growth[18].

The consequences and risks for the conceptus can be either immediate (of a physical nature in the form of spontaneous abortions [miscarriage], premature birth[19]) or long term (of a psychosocial nature with impact on the teenager's children). Due to the difficulty of the teenage mother to adapt to her new condition, she may decide to abandon the child and offer the baby for adoption. The newborn is more likely to be subject to bad treatment[2] and present a low birth weight[20]. Among the psychosocial consequences, interruption of studies and career opportunities (professionalization) are particularly worrisome. It is common to drop out of school and then have great difficulty when trying to resume studies. Different researches have indicated a high incidence of less school education and little professionalization among pregnant teenagers[21]. It is believed that, in addition to the problems already mentioned, there are other more severe problems of a psychological nature since, due to immaturity and emotional lability, important psychological changes could take place that will make adaptation to her new condition extremely difficult, thus worsening feelings that are already present prior to the pregnancy itself, including anxiety, depression, and hostility[22].

With regard to the father, he is usually 2–3 years older than the mother. Early parenthood is associated with increased school dropout rate, underemployment, reduced career opportunities, greater number of children, and greater divorce rate[2,23]. Taking into consideration all the factors mentioned hereinabove, pregnancy during teen years has indeed become a subject of concern, and studies are being conducted by specialists in order to reduce its occurrence. This challenge should be dealt with by society as a whole as the relevant solutions involve scientific, social, and political-economic resources. However complex this issue may be under many aspects, it is not to be treated only as a "problem" or a "disaster" in the life of teenage mothers. Many Brazilian women begin their reproductive life in the teen years, frequently as a result of cultural aspects and this does not necessarily constitute a "problem". On the other hand, society and government should provide services that consider the reproductive and sexual health of women in all phases of her lives.

This study intends to evaluate sexual activity and socioeconomic characteristics of pregnant teenagers and their partners to whom assistance was provided by a university hospital, with emphasis on epidemiologic aspects for development of appropriate preventive actions.

## METHODS

A retrospective analysis was made with regard to 152 pregnant teenagers who received global health care for pregnant teens at the Department of Pediatrics at the Santa Casa de Sao Paulo (SCSP) General Hospital during the January 01, 2002 to February 28, 2004 period. Individual interviews were made when the pregnant teenagers arrived at the hospital and their individual data were evaluated, including age, school years, age at first intercourse, method of contraception used, condom use, planned or unplanned pregnancy, abortion, drug addiction, matrimonial situation of the parents of the pregnant teenager, family reaction to her pregnancy, and presence or not of a role model of pregnancy in teen years in the family.

Additionally, data were also analyzed with regard to the partner of each pregnant teenager, including age, school years, work and income, if he fathered children with other women, criminal record, use of legal or illegal drugs, and also whether or not he welcomed the pregnancy and if he provides support to the pregnant teenager.

The research was evaluated and approved by the Committee on Ethics of Research Involving Human Participants at the SCSP. For analysis of data, the software used was Epi Info 6.0, including testing of differences between proportions (Chi-Square Tests) and Average Tests.

## RESULTS

The pregnant teenagers' ages at the time of conception ranged from 11–19 years, averaging 15.7 years. The average school years attended was 8.1 and 40.1% of the teenagers had dropped out of school; however, 75.4% of them had done so prior to becoming pregnant.

The average age of menarche was 11.75 years and the lowest age was 9 years. The age of sexual initiation of the teenagers analyzed was 14.2 years and the average period between sexual initiation and pregnancy was 1.4 years. Most teenagers, 65.3%, got pregnant from the first partner, 27.2% from the second, and 7.5% had more than three sexual partners; 19.9% got pregnant in the first year after sexual initiation, 38.4% in the second year. Only 35.7% of the teenagers mentioned use of some contraceptive method, 13.2% said that condom was always used, and 29.9% declared they never used any contraceptive method. However, only 14% had planned the pregnancy and 75% mentioned their pregnancy was not wanted. As to abortion ideation, 31.2% thought about the possibility of abortion and the average age of those teenagers was 13.6 years (total average of 15.7 years); 20.6% stated that if they had the means, they would have had an abortion; and 10.4% made at least one abortion attempt. With regard to the use of legal and illegal drugs, 16.9% were tobacco users and 6.7% were alcohol drinkers. We noted illegal drug consumption in 6.8%, of which in 81.8% of the cases, the drug was marijuana.

Of the 138 pregnant teens that answered about the civil status of their parents, we collected information that 35.8% of them were married, 32.1% were divorced, 12.4% cohabited without formal marriage, and 19.7% of the parents fit into other categories (dead, abandoned, etc.) (see Fig. 1).

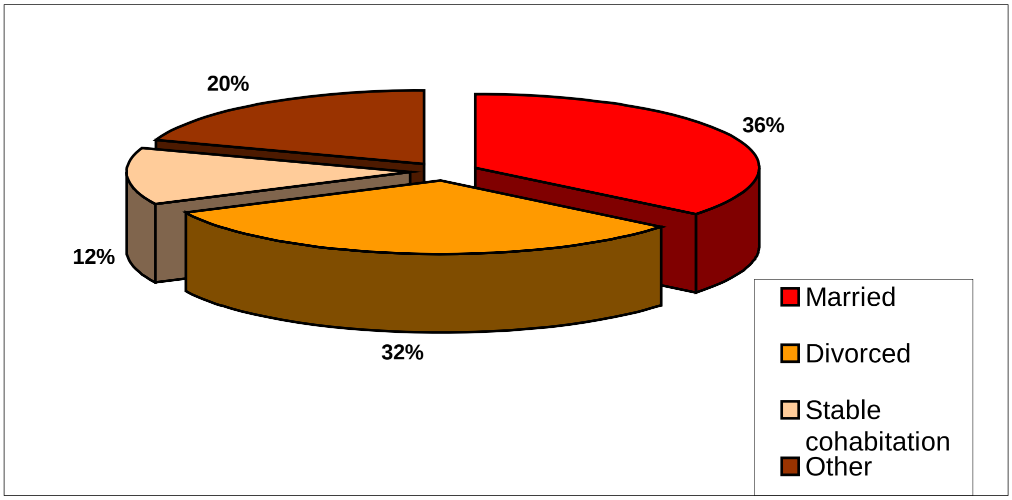

**FIGURE 1.** Characterization of civil status of the parents of pregnant teens.

We observed that 30.7% of the interviewed teens lived with their parents, 68.1% did not have a father permanently at home, while 33.7% had no father at all and 28.2% resided with their partners. In 54.8% of the cases, the family's reaction to the pregnancy news was negative. In 71%, there was a role model in the family of a previous teen pregnancy, i.e., the mother in 35% of the cases, a sister in 26.2%, and a cousin in 28.2%.

The partners' ages varied between 16 and 45 years with 84.0% younger than 25 and their average age was 21.9 years. The average schooling found was 8.5 years, however, we observed that 81.7% were school dropouts, with 25.8% of them having dropped out 1 year ago or less, and 47.5% dropped out of school more than a year ago. Their average school dropout time (time out of school) was 3.6 years. Most of them, 86.5%, were employed with an average income of $237.60 US (almost twice the minimum salary in Brazil); 8.9% had criminal records; 11.8% were alcohol users, 30.8% smoked. It was noted that 16.1% consumed illegal drugs and 85.8% of them were marijuana users. Most of them, 88.1%, will register the baby and 70.1% intend to live with or already reside with the teen mother; 50.0% mentioned happiness or enjoyment in connection with the pregnancy, 48.6% wanted the pregnant teen to get pregnant; 20.6% thought about abortion, and 19.1% mentioned having fathered another child with another woman.

We divided the pregnant teens in two groups according to age (Group 1, initial adolescence, ages between 10 and 15 years; Group 2, final adolescence, ages between 16 and 19 years) and compared data including schooling, pregnancy aspiration, use of contraceptive methods, and abortion ideation. We observed statistically significant differences ($p < 0.05$) relevant for this study; among the variables: pregnancy aspiration, use of contraception methods, and abortion ideation (Table 1).

**TABLE 1**
**Trends of Some Variables in Connection with Schooling, Pregnancy, and Abortion Based on Age Group**

| Variables | 10–15 Years, (%) | 16–19 Years, (%) | *p* Value* |
|---|---|---|---|
| Studies | | | |
| Yes | 52.6 | 64.2 | $p < 0.05$ |
| No | 47.4 | 35.8 | $p = 0.06$ |
| Wanted to get pregnant | | | |
| Yes | 24.3 | 36.9 | $p < 0.05$ |
| No | 75.7 | 63.1 | $p < 0.05$ |
| Used contraceptive method | | | |
| Yes | 29.6 | 39.3 | $p < 0.05$ |
| No | 70.4 | 60.7 | $p < 0.05$ |
| Abortion ideation | | | |
| Yes | 38.5 | 27 | *p* <0 .05 |
| No | 61.5 | 73 | — |

* *p* Value for Chi-Square computed for both age groups.

## DISCUSSION

The analysis of sexual activity factors and their socioeconomic characteristics regarding pregnant teens and their partners proved to be useful, as they are rarely investigated in the scientific literature that more frequently analyzes the adverse biologic impacts of early pregnancy. In recent decades, there has been an improvement in the educational level of the population as a whole, mainly among women and younger people. However, approximately 41% of the pregnant teens or teen mothers have less than 4 years education up to 4 school years, while among other young girls, only 21% have similar low schooling. So more school years are observed in the group of teenagers that still did not start reproduction; 28% against 9% (in the 9–11 school years category)[21]. In our research, the average school years was 8.1, a high rate when compared to the national average of 5.4 school years[25].

When we stratify average school years by age groups: 11–14 years, 15–17 years, and 18–19 years, we obtain the following results: 6.4 years; 8.6 years, and 7.4 years, respectively (see Fig. 2).

A comparison with the same stratification by age groups during research accomplished in Brazil in years 1992 and 1999 (see Fig. 3) shows that our average rate has remained higher in the three relevant groups.

This result was already expected since the pregnant teens of our sample resided mainly in urban regions. Data from the 2000 IBGE Census[25] confirmed that young people in more developed urban centers have greater access to school and, therefore, were above the national average; our pregnant teens sought specialized prenatal assistance themselves.

In spite of their higher schooling, the analyzed teenagers presented a 40.1% school dropout rate, which was high when compared to Werner-Wilson[10] or Leal et al.[26], whose published rates were around 30%; unfortunately most of them did not return to school[26]. With global health care for pregnant teens, 46.9% were studying within 2 years after childbirth[27]. Of those who dropped out of school, 75.4% of the 40.1% did so before the pregnancy, which indicated school dropout rates as a risk factor for precocious pregnancy. It is considered that teenagers that drop out of school generally do not complete professional qualifying programs, so it is presumed they will have inferior job opportunities. On the other hand, our study demonstrated that 24.6% dropped out of school after pregnancy and, as mentioned in Leal et al.[26], one of the reasons that could lead pregnant teens to drop out of school would be the fear of discrimination, as the evidence of active sexual life in teen years is a fact still not well accepted by our society in general.

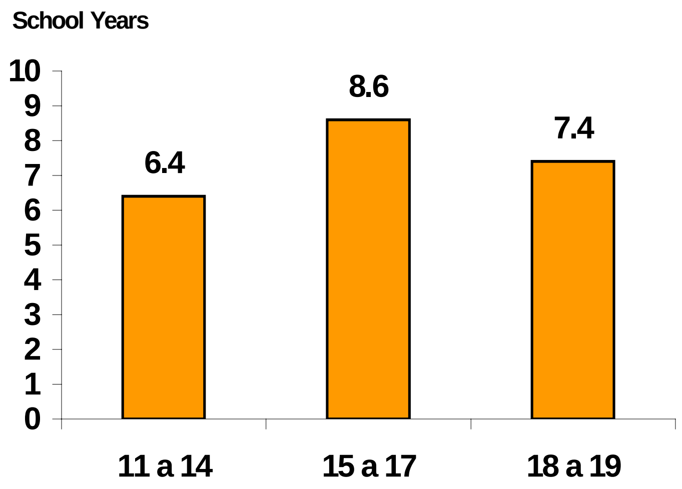

**FIGURE 2.** Average school years of pregnant teens attended at the Pediatrics Department at the University Hospital in Sao Paulo City by age groups.

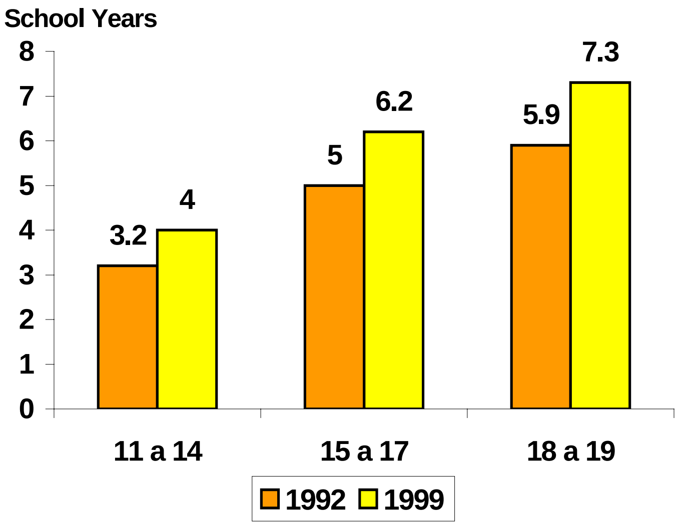

**FIGURE 3.** Average school years of 10-year-old and higher or older people by age groups. Brazil – 1992/1999. Source: 2000 Summary of Social Indicators. IBGE, 2001.

Among biological factors, it was observed that menarche had moved ahead approximately 4 months per decade in our century and its occurrence was now within the age range of 11–15 years[2]. In our study, the average menarche age was 11.75 years.

The decade of 1990 surprised the specialists with the rejuvenation of the fecundity in the country. While the rates declined in all of the age groups, between 15 and 19 years, there was a growth of 25%. The National Research about Demography and Health (DHS) of 1996[21] showed that the medium age for the first sexual relationship was 19.5 for women and 16.7 for men. More recent data[8] showed that youths were beginning sexual life much earlier; it varied between 13.9 and 14.5 years in the masculine sex and among 15.2 and 16 years among the women. The average age of our studied adolescents' sexual initiation was 14.2 years with an occurrence of time between sexual initiation and pregnancy of 1.4 years; the majority of the youths, 65.3%, were pregnant from their first partner.

Most of the adolescents began their sexual life without being appropriately protected by a birth-control method. The rates were larger among the younger ones who had less education and belonged to a lower socioeconomic class. Data[21] have shown that only 14.7% of the youths between 15 and 19 years used some birth-control method. We found larger rates of 29.6% between 10–15 years and 39.3% among 16–19 years, values conflicting when we analyze that only 24.3% of the adolescents between 10–15 years and 36.9% among 16–19 years wanted the pregnancy. Young people frequently tend to adopt a "magic thought" about risks related to the exercise of sexuality, not establishing in a conscious way the bond of sex with the pregnancy and with need for protection. In general, youth know about the existence of preventive methods, but they resist using them for fear that they can harm their health, because of ignorance of how to use them, difficulty in obtaining them, or even a conscious or unconscious desire to become pregnant[28].

Only 35.7% of the appraised teenagers used a contraceptive method and 29.9% never used a condom during sexual intercourse, even though most of them resided in urban centers (where there was greater access to school, better-quality health services, and media information) and presented a good schooling rate, which theoretically would reflect greater knowledge of contraception methods.

The utilization of contraceptive methods may not occur in an effective manner in this age range due to psychological factors inherent to adolescence, such as denial of the possibility of pregnancy and the belief that for occasional sexual intercourses, the regular use of oral contraceptives would not be justifiable. Furthermore, possession of oral contraceptives would be formal evidence of an active sexual life that would entail a new life stage where additional responsibilities would have to be assumed.

With regard to pregnancy aspiration and planning, this study confirmed results found by Camarano[29]. The great majority of pregnant teens, 75%, did not want to become pregnant and 86% had not planned the pregnancy. The unwillingness to get pregnant expressed by most pregnant teens exposes a lack of family planning that is quite common in this age group. However, it is a mistake to assume that all teenage pregnancy is unwanted, as many teenagers seek in pregnancy a change in their lifestyle, without, however, taking into account the future consequences of precocious pregnancy. In our study, 25% of the teenagers wanted pregnancy and 14% had planned it.

In spite of its illegal status, the abortion option seems well disseminated among teenagers. It is estimated that in Brazil, 1 million clandestine abortions are performed each year. According to data from the Ministry of Health, the provoked abortion constituted the fifth largest hospitalization cause between youths and the third cause of maternal death in the country; 16% among 15–24 year-old women in the poor areas from Brazil[5]. We observed that 31.2% of the appraised teenagers considered the possibility of abortion, 20.6% said that if they had proper means they would have had it, and 10.4% attempted abortion at least once. The high frequency of abortion could be the result of negotiations between the partners assuming the child/pregnancy[30]. However, university girls considered it to be more tolerable to deal with unplanned pregnancy through abortion because in middle class, it can be performed under more acceptable medical conditions, however constraining, clandestine, and expensive.

With regard to use of illegal drugs, 16.9% of the teenagers were tobacco users, 6.7% were alcohol drinkers, and 6.8% already had used or regularly used illegal drugs. These data are similar to those found by Carlini et al.[31]; 16.2% of the teenagers were tobacco users and 6.7% were alcohol drinkers in the same age group. Regardless of the said rates of utilization of legal or illegal drugs being considered relatively low, we believe that the sheer presence of the attitude deserves attention at prenatal planning in the age range studied herein.

The influence of the family on the sexual behavior of teenagers has been analyzed under various viewpoints, as the family context has a direct relationship with the time when sexual activity is initiated. Several studies[9] indicated that the single parent family, more commonly constituted by a single mother, was associated with earlier initiation of sexual activity and teenage pregnancy, even when other factors including family income were taken into account. Markham et al.[32] stated that the circumstance leading to teen pregnancy in single families was the fact that the mother was anxious due to her own feeling of frustration for having been abandoned by her partner, which was worsened by the economic burden of the family, which had a direct impact on passing her own hostility to the mother-daughter relationship and caused increased difficulty of communication between mother and daughter. In this situation, the teenager reacted by attempting to solve the conflicts out of her home, which was when she made an effort to maintain a steady relationship with her boyfriend. The sexual relations and consequent pregnancy were, in many cases, the result of her endeavor to be accepted and to find love and affection. In those cases, sexual pleasure did not appear to be the main reason for the sexual intercourse[10].

The parental marriage status of the appraised teenagers showed that most of them come from unstructured families and families with an absent father; 32.1% of the teenagers' parents were divorced and 68.1% of them did not have their father at home. Families well-structured and present were considered by Markham et al.[32] as a protective factor against precocious sexual initiation.

Negative family reactions to pregnancy were more evident among pregnant teens devoid of consensual union[13,14]. In our sample, we observed that in 54.8% of the cases, the family's reaction to the pregnancy news was negative. It is worth mentioning that the acceptance of the pregnancy by the family may be negative in the beginning, however, it tends to improve in due course, particularly because of the pregnant teens' need of support[15,34].

Abdallah et al.[33] mentioned that most pregnant teens had a mother or a sister that also became pregnant in their teens (role model), which was the (unconscious) psychological phenomenon of repeating the mothers' history. In our study, the presence of a role model occurred in 71% of the cases; the mother in 35%, sister in 26.2%, and cousin in 28.2%. In most reviewed literature, the topic of teen pregnancy was focused on problems based from the feminine point of view, where maternity and pregnancy and teen pregnancy were often synonymous terms[24]. The lack of study on partners of the pregnant teens ultimately and incorrectly emphasizes the belief that reproduction and its control would be "the exclusive responsibility of women", and did not include men[23].

However, the approach from a male point of view opens the possibility to access the reasoning about contraception, family formation, paternity assumption, etc. In our study, we confirmed that the partners of pregnant teens were on the average 6.2 years older than their partners, averaged 8.5 school years, and had an 81.7% school dropout rate; also, 8.9% of them had a criminal record. Vitalle[2] showed the high rate of school dropouts among partners of pregnant teens and on the average they are 2–3 years older than their partners. We also observed that 86.5% of them were employed with average income of $237.60 US. These data were consistent with reviewed literature and confirmed that even though being employed, the partners of pregnant teens were subject to positions not up to their qualification and of low pay[2]. The majority of the partners was involved with the adolescent mother; 88.1% intended to register the baby and 70% planned to live with or was already living with the partner; 50% demonstrated joy or happiness with the pregnancy.

## CONCLUSIONS

Among the analyzed pregnant teens, we found:

- Unprotected, precocious sexual initiation with low use of contraceptive methods, primarily condoms; among younger pregnant teens, the use of contraceptives was even smaller while abortion ideation was greater.
- Pregnancy frequently occurred in the first year of sexual life, which indicated necessity of early orientation.
- Generally, the teenager knew contraceptive methods, but resisted using them for fear of being harmful to her health.
- As to indication of ways for new research, we would like to emphasize the importance of the family and the perspective of global assistance to teenage girls; when appropriate initiatives are taken, we believe it is possible to help adolescents to engage in responsible sexual activity
- Education and active participation can help adolescents to get to know themselves better and lead to responsibility for their own sexuality, including decisions about contraception.
